# Fixing tacks induced bladder erosion and recurrent stones following laparoscopic inguinal hernia repair: a case report

**DOI:** 10.1186/s12893-020-00818-4

**Published:** 2020-07-21

**Authors:** Wan-Zhang Liu, Jun-Hai Qian, Zhi-Jiu Shen, Bin-Bin Yang, Yue Cheng

**Affiliations:** Department of Urology, Ningbo City First Hospital, the Affiliated Hospital of Ningbo University, #59 Liuting Street, Ningbo, 315010 Zhejiang Province China

**Keywords:** Case report, Mesh erosion, Complication, Laparoscopic inguinal hernia repair, Fixing tacks

## Abstract

**Background:**

Hernia mesh erosion into the urinary bladder is a rare complication of hernioplasty, and mesh immigration is the most probable pathophysiology. There is no report describing mesh erosion induced by fixing tacks in inguinal hernia repair.

**Case presentation:**

A 37-year-old man was admitted to our hospital with frequency, urgency and odynuria for 3 months. He received open right inguinal hernia repair in September 2014, and right laparoscopic hernioplasty for recurrence of the inguinal hernia in May 2015. In February 2019, he underwent a day-case transurethral cystoscopic operation for urethral and bladder stones. Cystoscopy revealed the existence of bladder stones and part of the eroded mesh on the right anterior wall, for which an open partial cystectomy was performed. The patient was followed up for 3 months postoperatively, during which no further mesh erosion or stone recurrence was detected by cystoscopy.

**Conclusion:**

This is the first case report describing mesh erosion into the urinary bladder by fixing tacks following laparoscopic inguinal hernia repair. In such a case, the eroded mesh and tacks need to be removed completely, but the effectiveness of a single transurethral procedure needs to be verified in more cases.

## Background

Hernia mesh erosion into the urinary bladder is a rare complication of hernioplasty, the underlying pathophysiology of which remains unclear and controversial. Although laparoscopic techniques show less postoperative pain and more rapid recovery than open surgery, individual cases of mesh migration such as bladder erosion have been reported following laparoscopic inguinal hernia repair [1]. To the best of knowledge, there is no report describing mesh erosion caused by fixing tacks. In this article, we report a case of mesh erosion into the urinary bladder by the fixing tacks, which was complicated by stone recurrence after the initial laparoscopic inguinal hernia repair. In addition, the relevant literature is reviewed.

## Case presentation

**Fig. 1:**
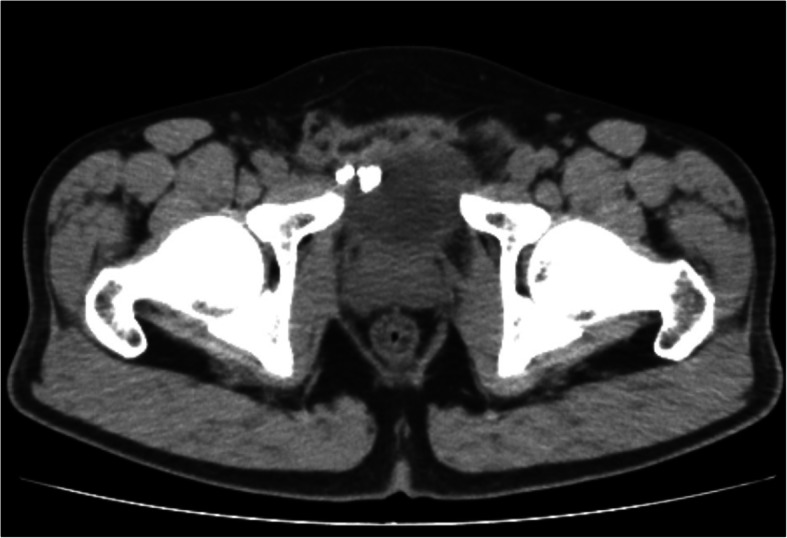
Abdominal plain CT-scan showing bladder stones and calcified tissue around the right inguinal region

**Fig. 2:**
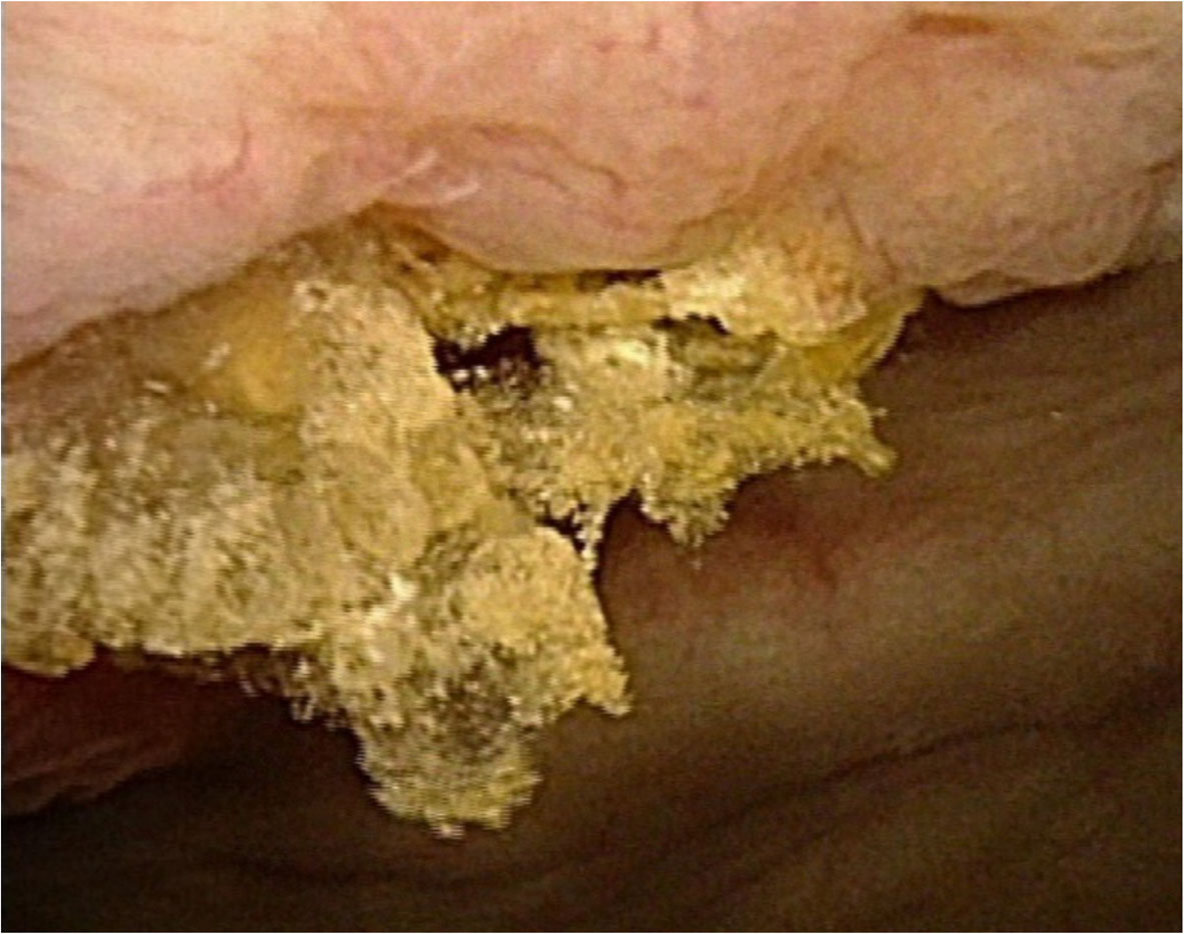
Cystoscopy showing bladder stones and part of the eroded mesh on the right anterior wall

A 37-year-old man was referred to our clinic in December 2019 with the chief complaint of recurrent frequency, urgency and odynuria for 3 months. Routine urinalysis showed 102 WBC/ul and 21 RBC/ul), but the result of urine culture was negative. Ultrasound of the urinary system revealed bladder stones attached to the anterior bladder wall and further abdominal CT-scan showed the existence of bladder stones and calcified tissues around the right inguinal region (Fig. 1). Subsequent cystoscopy demonstrated bladder stones and part of the eroded mesh on the right anterior wall (Fig. 2). The patient received open mesh repair for right inguinal hernia in September 2014, which recurred 9 months after the previous surgery, for which a TAPP repair was performed in May 2015. According to the second operation, a hernial orifice about 3 cm was identified on the right inguinal region, for which a 6 cm × 13 cm polypropylene mesh (COOK, USA) was applied and fixed with four non-absorbable metallic tacks on the dorsal side of the Cooper’s ligament and the pubic bone. In February 2019, the patient came to our clinic primarily with dysuria and hematuria for 2 days. CT scan identified urethral and bladder stones, for which a day-case transurethral cystoscopic holmium laser lithotripsy was performed. During the fragmentation, we found that the mesh was partially eroded and therefore cut the eroded portion by holmium laser under cystoscopy.

**Fig. 3:**
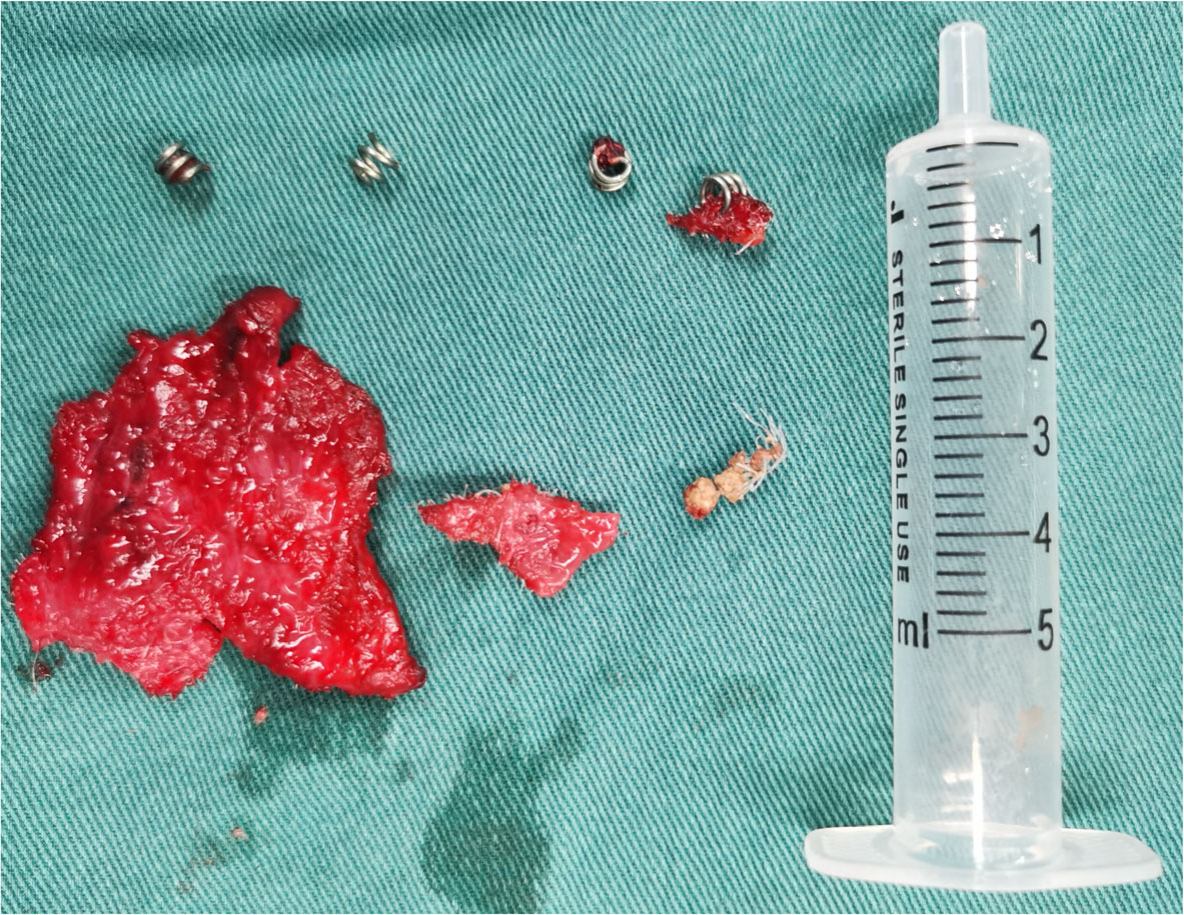
The eroded mesh and four fixing tacks, two of which have eroded into the bladder

For the recurrent bladder stones caused by the residue mesh, an open surgery was performed to remove them through a 7-cm incision along the midline of the lower abdomen. The rectus abdominis was dissected bluntly; the linear alba was incised straightly; and the peritoneum was separated and pushed upward to expose the anterior wall of the bladder. Under the excellent exposure of the field, a tough tissue was touched around the anterior bladder wall. By nipping the tissue with forceps, we dissected into the bladder with scissors and forceps. At the end of the tissue where the eroded mesh and bladder tissue were located, we found stones and four tacks attached to the dorsal side of Cooper’s ligament and the pubic bone. After taking off these four tacks, we noticed that two of them were eroding into the bladder (Fig. 3). The bladder defect was closed in two layers with 2–0 suture. Finally, the bladder was inflated from the indwelling urethral catheter and demonstrated no leak. A closed drainage was indwelled in the preperitoneal space. A urinary catheter was inserted and retained for 3 weeks. No mesh residue or stone recurrence was found by cystoscopy during the 3-month follow-up period.

## Discussion and conclusion

Mesh reinforcement through either an open or laparoscopic approach is the most common procedure for inguinal hernia repair. However, improper usage of the mesh, including improper selection of the mesh size and inappropriate placement of the mesh, may cause recurrence of inguinal hernia. Compared with open surgery, laparoscopic repair can provide better exposure of the complete surgical field and is less likely to induce postoperative complications such as infection and bleeding. A retrospective study by Liu et al. [2] demonstrated that endoscopic repiar of recurrent inguinal hernias was better than other techniques, especially in cases with deep adhesion and scarred or fibrotic tissues. And a different approach may avoid anatomy variation due to the first operation.

There are still controversies over whether the hernia repair mesh should be fixed during laparoscopic surgery. Some studies reported that for cases with a nonrecurrent uncomplicated hernia defect < 3 cm, non-fixation could offer a therapeutic efficacy comparable to that of mesh fixation with a shorter operative time, less short-term postoperative pain, a lower risk of urine retention, and fewer costs. But a higher risk of recurrence has also been the main concern among surgeons. Lo et al [3] found that the recurrence rate of inguinal hernia in mesh non-fixation group was higher than that in fixation group, though the difference was not statistically significant (0.53% vs. 0.14%, *p* = 0.28). Few studies have concluded that mesh fixation by either chemical glue or tacks for recurrent inguinal hernia has satisfied outcomes [2]. In the present case, tack fixation was performed due to the big size of the recurrent hernia defect. No sign of inguinal hernia recurrence was noticed during the 5-year follow-up period after the previous hernia repair procedure in 2015.

Mesh erosion into the urinary bladder is a rare complication and mostly reported as a case series. It may occur 3 months to 20 years post-operation [4]. Mesh migration and foreign body-induced reactions have been deemed as a probable pathophysiology [5]. In this case, we found invasion of two tacks with the eroded portion of the mesh into the anterior bladder wall, which we suppose is due to the mild but chronic injury and inflammation caused by the tacks.

Partial or complete removal of the mesh has proved to be safe and effective treatment for the management of mesh erosion. Most of these procedures are performed through an open approach. Nikhil et al. [1] presented a case of bladder erosion and stones after laparoscopic hernia meshplasty, and managed the problem successfully through transurethral cystoscopic partial mesh excision and extraction. Although our patient had been treated with the transurethral cystoscopic procedure, a secondary open surgery was still needed in 10 months. We believe that the eroded mesh residue and tacks played an important role in the recurrence of bladder stones.

To the best of our knowledge, this is the first report describing mesh erosion caused by fixing tacks. But with wider application of the laparoscopic technique in clinical practice, it cannot be the last case of mesh erosion caused by fixing tacks and therefore deserve attention of surgeons concerned. Once identified, the eroded mesh and tacks should be removed completely, with or without partial cystectomy. As this is only a single case reported herein, the effectiveness of a single transurethral procedure needs to be further verified in more cases.

## Data Availability

Not applicable.

## Abbreviations

CT: Computed tomography
WBC: White blood cell
RBC: Red blood cell
TAPP: Transabdominal preperitoneal prosthetic
